# Overview of natural hydrogels for regenerative medicine applications

**DOI:** 10.1007/s10856-019-6318-7

**Published:** 2019-10-10

**Authors:** Marta Calvo Catoira, Luca Fusaro, Dalila Di Francesco, Martina Ramella, Francesca Boccafoschi

**Affiliations:** 1 Department of Health Sciences, University of Piemonte Orientale, 28100 Novara, Italy; 2 Tissuegraft srl, 28100 Novara, Italy; 3 Center for Translational Research on Autoimmune & Allergic Diseases - CAAD, Novara, 28100 Italy

## Abstract

Hydrogels from different materials can be used in biomedical field as an innovative approach in regenerative medicine. Depending on the origin source, hydrogels can be synthetized through chemical and physical methods. Hydrogel can be characterized through several physical parameters, such as size, elastic modulus, swelling and degradation rate. Lately, research is focused on hydrogels derived from biologic materials. These hydrogels can be derived from protein polymers, such as collage, elastin, and polysaccharide polymers like glycosaminoglycans or alginate among others. Introduction of decellularized tissues into hydrogels synthesis displays several advantages compared to natural or synthetic based hydrogels. Preservation of natural molecules such as growth factors, glycans, bioactive cryptic peptides and natural proteins can promote cell growth, function, differentiation, angiogenesis, anti-angiogenesis, antimicrobial effects, and chemotactic effects. Versatility of hydrogels make possible multiple applications and combinations with several molecules on order to obtain the adequate characteristic for each scope. In this context, a lot of molecules such as cross link agents, drugs, grow factors or cells can be used. This review focuses on the recent progress of hydrogels synthesis and applications in order to classify the most recent and relevant matters in biomedical field.

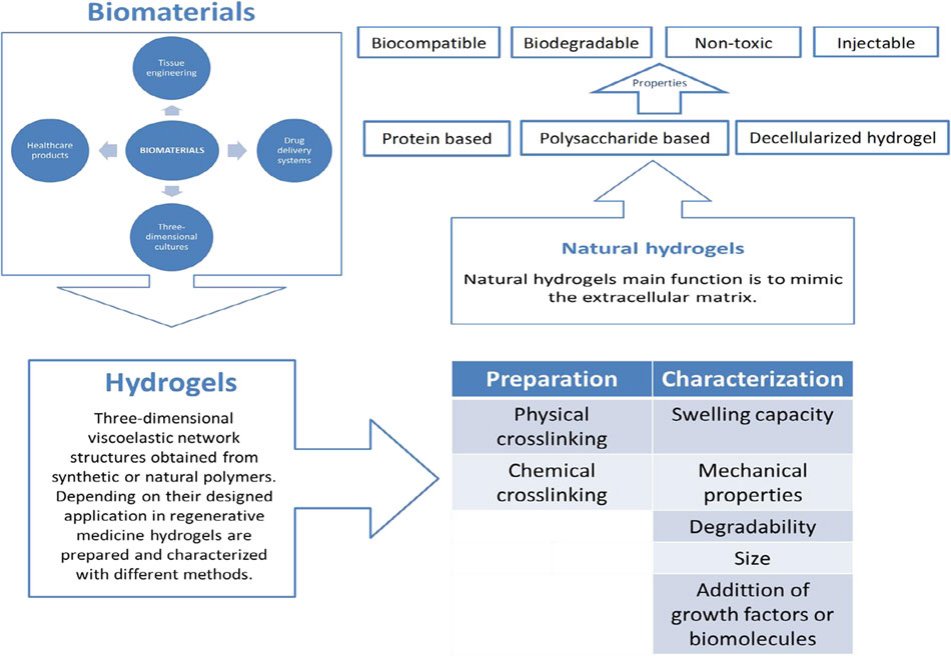

## Introduction

Biomaterials have been a great matter of interest in the past years, they are already been used in many biomedical fields and continue to be studied worldwide as there are countless types and represent a great potential in the achievable applications. They are described as “any substance or combination of substances, of natural or synthetic origin, which can be used on a clearly defined time period as a whole or a part of a system that treats, speeds healing or replace tissues, organs or a function of the human body” or as “any substance that has been engineered to interact with biological systems for a medical purpose”, therefore they offer a possible solution as scaffolds, drug delivery systems, cell culture systems and biological substitutes for tissue engineering. Among biomaterials we find a type of three dimensional (3D) polymers called hydrogels [1].

The term hydrogel describes 3D network structures obtained from a class of synthetic and/or natural polymers which can absorb and retain significant amount of water. Hydrogels are swollen 3D viscoelastic polymeric networks which have similar physical properties to natural tissue. The network is formed by crosslinking polymers with covalent bonds or noncovalent interactions and their structure can be then designed to suit the final application. Their unique ability to absorb and retain water is given by their hydrophilic nature, the amount of water they absorb depends on factors like the hydrogel structure, the crosslink density, the composition of the solution and the technique used to synthetize it. There are various types of hydrogels, which can be classified according to the materials used to synthetize them, giving natural or synthetic hydrogels, or according to the methods used to synthetize them, which can be physical or chemical crosslinking. Hydrogels in general have many characteristics which make them an interesting matter of research: they have a high versatility due to the fact that their chemical and physical properties can be manipulated to design a gel most suitable for the required application. Hydrogels unique ability to form swollen 3D networks gives them the possibility to diffuse molecules and cells, nonetheless their similarity to natural soft tissue is also of great interest for biomedical applications [2]. Natural hydrogels, which this review will be focusing on, include collagen, silk fibroin, hyaluronic acid, chitosan, alginate and hydrogels derived from decellularized tissues. Their unique properties include: biocompatibility, biodegradability, low cytotoxicity, the possibility to tailor the hydrogel into an injectable gel and their similarity to physiological environment. However, natural hydrogels do have some limitations, for example they do not have strong mechanical properties and are not easily controllable due to their batch-to-batch variation. For these reasons, natural hydrogels are often combined to synthetic ones, creating composite polymers, and are still widely experimented [3, 4].

The possible employments of hydrogels are a very wide topic which still has a lot to offer, the range goes from domestic use to biomedical applications. Hydrogels in general have been extensively studied for different biomedical applications, which include drug delivery systems, 3D cultures, tissue implant, tissue regeneration, contact lenses and use in healthcare products. Every different type of hydrogel can be tailored to suite the application it is designed for, therefore hydrogels will be made using different techniques to give them the necessary chemical and physical properties.

## Hydrogels preparation

Hydrogels are usually prepared using hydrophilic monomers in order to form a crosslinked network that can absorb water. The gelation phenomenon happens when the polymer mixture goes from a sol state to a gel state, this is called sol–gel transition and the gel point can be calculated for each hydrogel using rheological studies [5].

There are many ways to synthetize a hydrogel, but these methods can be divided in two major groups: chemical crosslinking or physical crosslinking. Chemical hydrogels can be covalently crosslinked and methods include: grafting, radical polymerization, click chemistry, enzymatic reactions, thermo-gelation and radiation crosslinking. Moreover, adding to the hydrogel precursor ions like Ca^2+^, Mg^2+^, Zn^2+^ can induce gelation through ionic bond formation in polymers such as alginate rich in anionic groups. However, naturally derived hydrogels are mostly formed by self-assembly physical crosslinking processes, which mainly include the change of intermolecular interactions such as ionic crosslinking, hydrophobic interactions and hydrogen bonded gels. All these processes are obtained modifying the temperature of the hydrogel precursor increasing up to 37 °C or drastically decreasing it (−20/−80 °C) [6]. However, during the gelation process many parameters can be changed or controlled in order to achieve the suitable hydrogel structure of interest [7]. The combination of chemical and physical crosslinking is also an option for hydrogel formation. Natural hydrogel’s gelation methods are listed in Table 1.

**Table 1:** Natural hydrogel’s gelation methods

Gelation method	Hydrogel origin	References
Physical methods		
Temperature increase	Chitosan	[78–80]
	Collagen	
Cryogelation	Silk	[81]
Chemical methods		
Crosslinkers	Chondoritin	[27]
	Hyaluronic acid	[82]
	Gelatin	[81]
Cation adding	Alginate	[83]

## Characterization

Depending on the application, hydrogels can be differently characterized in swelling, size, mechanical properties and their degradation rate.

From the point of view of their mechanical properties, the hydrogels are characterized by the elastic modulus which exhibits a pronounced plateau extending to times at least of the order of seconds, and by a viscous modulus which is considerably smaller than the elastic modulus in the plateau region [8]. For this reason, hydrogels are considered unique viscoelastic materials, as they possess both properties. Their mechanical strength depends on the crosslink density, however the stronger the gel the less it can absorb water.

The degree of swelling must be determined and characterized; this parameter is restricted by the elastic forces in the gel, this means that the hydrogel has infinite solubility which is restricted by the elastic forces of the network. The equilibrium swelling degree and the elastic modulus of hydrogels depend on the cross-link and charge densities of the polymer [9]; the amount of water absorbed by the gel depends on the porousness of the structure, the type of materials used and the crosslink density. Hydrogels may exhibit drastic volume changes in response to specific external stimuli, such as the temperature, solvent quality, pH and/or electric field; this is the reason why hydrogels swelling capability need to be studied in detail for their designed application.

Finally, the degradation kinetics of each gel must be well characterized and tracked. This can be done by labelling hydrogels with fluorophores. As mentioned earlier this parameter must be characterized according to the hydrogels’ designed application [10], as this property alone can significantly change the hydrogels’ characteristics [11].

In this review we will be focusing on the types of materials used to synthetize natural hydrogels, their chemical and physical characteristics, their limitations, and finally their possible applications.

## Natural hydrogels

Naturally derived hydrogels can be classified in 3 groups: protein-based materials, polysaccharide-based materials and those derived from decellularized tissue. Natural gels are typically formed of proteins and extracellular matrix (ECM) components, this makes them inherently biocompatible, bioactive and possibly suitable for many biomedical applications as they promote many cellular functions. As said earlier, their structure and properties resemble the native soft tissues, however they do have limitations, mostly the difficulty in manipulating them, as they have a high batch to batch variation.

**Fig. 1:**
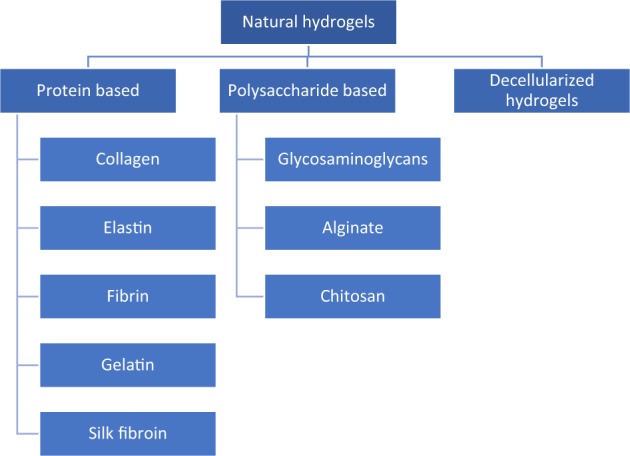
Natural hydrogels materials classification. Protein-based materials

Each type of hydrogel possesses different characteristics which makes it more suitable for the application intended for it, therefore we will look at the different types in detail. Figure 1 summarizes the different natural materials used to produce hydrogels.

These types of hydrogels include ones derived from collagen, gelatin, elastin, fibrin and silk fibroin. Elastin, collagen and fibrin are widely found proteins in the ECM structure, giving it the required strength and elasticity to function properly, making them very promising materials for tissue engineering and cell culture systems. The source of these proteins is mainly animal extraction, collagen for example is mainly extracted from porcine tissue or from murine tails, meanwhile fibroin is found and extracted from insects [12].

## Collagen

Collagen is the main ECM protein in the body and its function is to provide mechanical support to contrast action forces in order to resist an eventual repetitive plastic deformation and, at the same time, the collagen type and fibers orientation defines the diverse cell types disposition in the tissue.

Collagen structure is defined by four different organizations levels. The primary structure is composed by the tripeptide sequence of –(Gly–X–Y–)n–, where glycine (Gly) constitutes about 30% of the total amino acid content in collagen and generally, X is a proline and Y is an hydroxyproline. The secondary structure is formed by amino acid triplet chain repetitions. The third organization level is a left triple helix where each chain contains around 1000 amino acids. Collagen fibers is the denomination for the quaternary structure of collagen and it is formed by self-assembly fibers [13].

Until now 29 types of collagen were individuated but collagen I is the most commonly used and researched. In native tissues collagen mechanical properties are ideal, however in collagen based biomaterials the mechanical strength is insufficient due to the lack of covalent crosslinking. For this reason, crosslinking by physical, chemical, and biological methods [14] were used in order to attempt an increase of the mechanical performances.

From the biological point of view, collagen presents low antigenicity, low inflammatory response, biocompatibility, biodegradability, and excellent biological properties [15–17].

Usually collagen I hydrogels are prepared by adding NaCl into the collagen solution in order to obtain better optical performance and mechanical properties, also, increasing pH around the isoelectric point at a low ionic strength can improve the linear viscoelastic properties and transparency [18]. Living cells and bioactive molecules can be added in the collagen solution before gelation.

Micro and macro structure of hydrogels are essential for a cell grow and consequently for tissue regeneration. In this context, gelation process parameters and 3D technology can mimic the specific morphology of the target tissue [19].

In some tissues, such as nerves and corneas, the alignment of collagen fibrils is important to guide the cell orientation and migration. With this purpose, some methods have been developed so as to create micropatterned templates, keratocytes were used [20] and by using magnetic nanoparticles and applying an external magnetic field it is possible to control the collagen fibrils orientation [21].

Collagen is used in various tissue targets due to its great bioperformances, for this reason a large number of studies were carried out, which include its combination with different synthetic materials and/or biomolecules using various fabrication techniques. These numerous studies are summarized in detail by Lin et al. [22].

The limitation in the use of collagen as a biomaterial is that the degradation product of collagen results in the production of amino acids that have thrombogenic potential and activate the coagulation cascade. In addition, the high cost of pure collagen curtails its utilization as a cost effective approach in large scale biomaterials use [23].

## Elastin

Elastin is an important connective tissue protein that provides elasticity to organs, its crosslink network is made of its soluble precursor tropoelastin, this results in an insoluble polymer. The elastic fibre is composed of amorphous elastin and microfibrils which act as a scaffold on which elastin is deposited. An important property of tropoelastin and elastin-like peptides is their potential to self-assemble under physiological conditions. Its most remarkable mechanical property is its elasticity, which has made elastin a material of interest when it comes to building scaffolds for skin substitutes and vascular grafts. On the other hand, high porous hydrogels made from elastin or elastin like materials are studied as 3D cell cultures, drug delivery systems and gene delivery systems. Since elastin is a natural component of the ECM it has some interesting properties which include biocompatibility and biodegradability. Although elastin is a very important protein for the ECM structure, it is not used as often as other proteins in hydrogel production; this is because the proteins used in biomaterials have to be pure, therefore elastin need to be purified from the elastic fibers that form during its synthesis. During this purification process, contaminations often take place which can then lead to immune responses by the body, furthermore elastin has a tendency to calcify [24–26]. In more recent years more successful elastin purification methods have been developed [27], however since elastin is highly insoluble its soluble forms are often used, like tropoelastin, alpha-elastin, elastin like polypeptides (ELPS) and a type of genetic-recombined elastin (ELRs). Elastin based hydrogels can be synthetized in various ways depending of the form used: use of glutaraldehyde as a crosslinking agent, click chemistry can be utilized by adding compatible functional groups [28], electrospinning and self-assembly crosslinking. During the synthesis process elastin’s mechanical properties and the hydrogels porosity can be altered to create the hydrogel of interest, an example is using high pressure CO_2_ during the fabrication to increase the pore size, this creates a highly porous hydrogel suitable for cell growth [29]. Different hydrogels can be created with elastin. In this context, a method can be adding elastin to other type of natural hydrogels to improve the elastic properties. This is particularly a matter of interest in creating vascular scaffolds, where collagen gels can be enriched with elastin [30]. Collagen–elastin gels have also been proven to be successful in creating scaffolds for skin wound healing [31]. In addition to this studies have also proven that elastin scaffolds enable and stimulate fibroblast growth [32]. Elastin can also be used in combination with gelatin to form Gelatin–Elastin based hydrogels, although no big significant changes were brought to the gel [33].

## Fibrin

Fibrin is a protein particularly involved in the natural repairing process of the tissues and in the coagulation cascade. Fibrinogen is the inactive form of fibrin and when activated it plays a key role in the wounded tissue regeneration by forming an extensive fiber network. In this context, fibrillar gel composed of fibrin clots provides support forming a scaffold which is involved in the final healing process.

Fibrinogen can be obtained from blood and it can be used as an autologous source for the scaffold, mitigating or eliminating the risks of immunological incompatibility [34]. These gels are considered an alternative solution to collagen due to the fact that they are easily obtained from patient’s blood [35]. Moreover, some studies report that cells grown into a fibrin gel produce more collagen and elastin than cells seeded into a collagen gel [36] and, when used for delivering cells (osteoblast) or to regenerate bone, fibrin gel obtains better results that autologous bone graft [37].

Fibrin based hydrogels are commonly used in cardiac tissue engineering, however the main obstacle is the low mechanical strength. Furthermore, the final material must support the contraction and cell orientation. Here, comprising aligned microthreads were distributed uniformly throughout a fibrin based hydrogel, with a significant impact in cell alignment and mechanical properties [38].

In this perspective, some studies combine fibrin with other molecules or materials in order to mimic the target tissue. Proteinase inhibitors, such as ε‐aminocaproic acid (ACA), were mixed to prevent the fibrin matrix degradation for bone tissue regeneration. Arun Kumar et al. used another approach which consists in a nano‐fibrin, calcium sulfate, and chitin mix to produce a hydrogel with a good performance in bone repair [39].

Another approach is the use of spray, phase‐inversion technique, and deposition to produce an hybrid material based on poly(ether)urethane and polydimethylsiloxane layered with fibrin containing growth factors, in this manner it is possible obtain a material with good mechanical proprieties and bioactive behavior [40].

## Gelatin

Gelatin is a natural and low-cost vascular polymer whose properties, such as minimal immunogenicity and appreciable degradability, make it one of the best options for tissue engineering. This polymer is obtained through denaturing the triple helical conformation of collagen, there are two types of gelatin derived from 2 different treatments: gelatin A processed by acids (pH 1–3) and gelatin B processed with alkaline solutions, type A being the most preferable for scaffold building. Gelatin has been also used as a coating agent to enhance cell attachment as a technique of vascular tissue regeneration [23].

Gelatin presents gel forming, thickening, emulsifying and foaming proprieties. The mechanical one’s depend on supramolecular structure [41].

Synthetization of anionic or cationic gelatin is based on extraction conditions and fundamentally for filling molecules through electrostatic interaction. Since gelatin stability at high temperatures and wide range of pH allows synthetic polymers to be grafted on gelatin backbone. Methods are “grafting from”, polymerization on the functional surface of the substrate, “grafting to”, grafting end functionalized polymer to the substrate, or “grafting through” [42].

Gelatin-based formulations are employed in biomedical and therapeutic molecule delivery. [43]. Polyethyleneimine functionalized gelatin can increase transfection efficiency thanks to positive nanoparticles that facilitates DNA loading [44]. It demonstrate the possibility to use hydrogel as vectors for gene delivery.

Methacryloyl group-grafted gelatin, also known as gelatin methacrylate (GelMA) had been used for 3D printing in microfabricated blood vessel. Gelatin electrospun fibers loaded with antibiotic drugs (ciprofloxacin) have a strong antibacterial activity but this gelatin has to be reinforced with materials such as graphene oxide and boron nitride, to improve poor mechanical proprieties. [45–48].

Using electrospinning processes to generate tubular scaffolds allows obtain similar morphology to the native ECM structure, gelatin copolymer behaved as a natural cell responsive surface under mechanical stimulation [49].

Panzavolta et al. obtained electrospun gelatin nanofibers cross-linked with genipin, a low-toxicity agent, which reduces the extensibility of the electrospun mats, producing appreciable improvements in elastic modulus and breaking stress. [50]. Long-term studies on cross-linked electrospun gelatin are still under study to confirm its efficacy as a material scaffold for blood vessel tissue engineering.

## Silk fibroin

Silk fibroin is a protein produced by silkworms, spiders and scorpions, even though it is mainly obtained from Bombyx mori silkworm. Its amino acid composition is 43% glycine, 30% alanine, and 12% serine [51].

Dissolved silk fibroin is possible by using different kinds of solvents like lithium bromide, formic acid, ionic liquids, and ternary solvent system CaCl_2_/ethanol/water. The previous necessary step consists on removing the sericin, an antibacterial and UV-resistant protein which glues the silk fibers, using degumming process via dilute sodium carbonate solution or urea, citric acid, and sodium oleate [52]. In order to stabilize the film produced with fibrin, silk I and II are combined. Silk I is water soluble, whereas silk II is an antiparallel beta-sheet conformation and is insoluble. Young modulus, strain and tensile strength are improved when silk I is converted into crystalline silk II though annealing process [53].

Because of its excellent mechanical properties, low adverse immune reaction, minimal thrombogenicity, and compatible degradation rates it is employed in vascular tissue engineering [23]. Furthermore, it is compatible with several manufacturing processes, such as electrospinning or gel spun.

3D printing technology employs silk hydrogel with different techniques extrusion, lithography and recently digital light process using a grafting methacrylate [54]. Silk is also combined with other biopolymers, such as gelatin and alginate to obtain the adequate rheology characteristics for the bioink [55].

## Polysaccharide

### Glycosaminoglycans

Glycosaminoglycans (GAGs) are long unbranched chains composed of disaccharide units containing carboxylic and/or sulphate ester groups. These functional groups bridge and link collagen to form the ECM network.

An example of GAG is hyaluronic acid (HA), present in the mammalian connective tissue where it acts as a lubricant. HA is an anionic non-sulphated GAG that consists of glucuronic acid and N-acetyl glucosamine [15]. HA has some remarkable properties such as hydrophilia, non-adhesiveness, biodegradability [15, 56], and the ability of being completely resorbable through multiple metabolic pathways [57]. Another advantage of HA is that its production is easy and controllable in a large scale through microbial fermentation [17], avoiding the risk of animal-derived pathogens. Moreover, HA can bind large amounts of water to form hydrogen bonds with the solvent.

There are studies that evidence that HA is involved in angiogenesis, ECM organization, wound healing, inflammation, and other biological processes [17]. The studies on graft based esterified HA show a lower axial strength and higher stiffness than normal porcine arteries [58]. Improvements on the creation of an endothelial monolayer when compressed are achieved through Hyaff-II, a biomaterial obtained from total esterification of hyaluronan with benzyl alcohol [17]. Porcine model implanted with Hyaff-II showed graft occlusion at the end of 3 months, however the graft almost completely degraded while the formation of neo-tissue was complete with endothelium, organized collagen, and elastin [59]. This scaffold allows neoangiogenesis and endothelialization of the luminal surface, it supports elastin synthesis without chemical and cellular preconditioning in vitro, for these reasons it is considered a very promising solution [17]. HA derivatives are used successfully as scaffold materials for chondrocyte growth [60], bone and skin tissue regeneration, for treating vascular diseases due to their properties of anti-inflammation and biocompatibility [61].

### Alginate

Alginate is a polysaccharide component of brown algae cell walls and some bacteria capsule [62]. Its structure is based on two different monomers: β-D-mannuronate (M) and α-L-guluronate (G) organized into blocks. Since G-blocks form ionic bridges, alginate mechanical properties are related with the amount and disposition of these blocks [63]. From the biological point of view for medical applications alginate represents nontoxic and noninflammatory properties, in vivo degradation does not occur, however cell adhesion is poor and mechanical characteristics are insufficient. Alginate applications were explored in different tissues such as liver, nerve, heart, and cartilage [64].

### Chitosan

Chitosan is a linear polysaccharide composed of β-(1–4)-linked D-glucosamine with randomly dispersed N-acetyl-D-glycosamine groups, obtained by partially deacetylating chitin, the primary structure of arthropods exoskeletons. Structurally chitosan is similar to glycosaminoglycans contained in the ECM. Physical and mechanical properties are directly correlated with chitosan molecular weight and degree of deacetylation.

The advantages of using chitosan in hydrogel preparations are: antibacterial properties, easy to sterilize, a low cost, bioactive, biocompatible and its degradability can be controlled by changing the level of deacetylation [65]. This type of hydrogel is easily affected by parameters such as pH and temperature. Among its disadvantages are its poor mechanical properties, however this problem can be overcome by crosslinking with chemical materials [12] or with gelatin [23].

Because chitosan has a stimulatory action on leukocytes, and antibacterial properties, thus conferring an enormous potential as a natural polymer for wound-healing applications [66].

Chitosan was studied as a vascular scaffold in combination with gelatin by Zhang et al. who fabricated a two layers tubular chitosan and gelatin scaffold. In order to improve the endothelial cells (ECs) and smooth muscle cells (SMCs) spreading and proliferation [67], chitosan was modified with dextran sulfate with a satisfactory results in vitro and in vivo [68]. To reduce the possibility of clot formation in the vessel, Madihally and Matthew designed a family of chitosan scaffolds which include heparin-modified porous tubes [69].

Given its controllable degradability and its short duration in physiological conditions chitosan is a very good candidate in the production of hydrogels applied as drug delivery systems [70].

Lastly, chitosan enhances bone formation, however, due to inadequate mechanical properties it has to be combined with other materials, such as alginate, calcium phosphate or HA in order to use it in the orthopedic field [54].

### Decellularized tissues

The goal of tissue or organs decellularization is to mimic native tissue and provide an environment properly recognized by cells, this is obtained by successfully removing cells while preserving the micro-and-macro architecture of ECM and the niche for cells.

In order to obtain an optimal decellularized matrix, many factors must be considered, including the tissue source, the preparation procedures and the decellularization efficacy. In addition, other factors such as the use of chemical cross-linking agents or final sterilization should be under control as they may affect the structure of the ECM [71].

Specific characteristics such as cell density, matrix density, geometry, tissue thickness, and shape should be considered to process it with the optimal decellularizing method. To preserve the matrix micro/nano-structure, the composition and the biological properties, the decellularization method should be selected rationally and it must be scientifically justified for each kind of matrix or organ [71]. The critical point is not only to use the optimal physical forces exposure, but also the choice of non-physiologic chemical and biologic agents such as detergents and enzymes.

The right choice of an optimal decellularization method and its efficacy is crucial for clinical success or failure. Equally important are the removal of cell remnants and chemical treatment trace in order to avoid a possible host immunological response [72].

In most soft tissues collagen and elastin preservation is crucial to maintain the necessary physiological elasticity. The collagen role is retaining the tensile strength, while the elastin fibers preserve the elastic properties of the scaffold and GAGs provide it the viscoelasticity [73].

ECM scaffolds present a multifunctional structure with the ability to induce cell attachment, proliferation, migration, differentiation and maturation, even stem cells [74], due to cell signaling.

Molecules such as growth factors, glycans, bioactive cryptic peptides and natural proteins like collagen and fibronectin, promote the biologic activities such as, cell growth, function, differentiation, angiogenesis, anti-angiogenesis, antimicrobial effects, and chemotactic effects. [71]. Indeed, many studies demonstrated that proteoglycans from the ECM are involved in this molecular events [75, 76]. This specific properties represent the advantage of utilizing decellularized materials instead of other biological or synthetic materials [74].

Hydrogels from decellularized tissues can be obtained by reducing dry material into small pieces, then digesting the pieces with collagenase and/or pepsin in acid media for different time intervals depending of the type of tissue. This enables the acquirement of a water-soluble, low-molecular-weight product biopolymer. The desired concentration of ECM is obtained by diluting the pregel before the gelation phase which normally occurs at temperatures of around 37 °C.

Optimizing hydrogels derived from decellularized tissues is not trivial, this is because of several reasons: they can derive from different tissues with a specific decellularization and digestion method, resulting in hydrogels with different ECM concentrations depending on the desired application. For this reason, Saldin et al. considered all these variables to create a detailed summarized overview of decellularized hydrogels [77].

To summarize the major aspects of producing a hydrogel, Table 2 quantifies the level of easiness in terms of obtaining the material from its original source, biocompatibility and its processing into a hydrogel.

**Table 2:** Comparison of source, biocompatibility and processing of the different biomaterials

Material	Source	Biocompatibility	Processing
Collagen	+	+++	++
Elastin	+	+++	+
Fibrin	+++	++	+
Gelatin	++	+++	++
Silk fibroin	+	++	+
Glycosaminoglycans	++	+++	+
Alginate	++	+++	+
Chitosan	++	+	+
Decellularized tissue	+++	+++	+

The score goes from one to three plus to indicate low, medium and high simplicity respectively

## Conclusions

To date, regenerative medicine generates very high expectations, which are not met by current technologies. For this reason, new technologies are rapidly evolving, in order to obtain adequate structures that can thoroughly mimic the complex environment found in the human body. The type of material to employ is a crucial choice that determines the achievement of excellent results. In this context, natural hydrogels offer a valid option. Within this group of innovative materials, hydrogels derived from decellularized tissue may provide further advantages, due to the fact that they represent “smart biomaterials” able to interact and adapt to the surrounding microenvironment and related functions.

Many aspects concerning mechanical properties, preparation and standardization of these biomaterials still need to be improved in order to enhance these products in clinical use.

## References

[1] Grumezescu AM, editor. Nanobiomaterials in soft tissue engineering: applications of nanobiomaterials. Amsterdam: William Andrew; 2016.

[2] Francis L, Greco KV, Boccaccini AR, Roether JJ, English NR, Huang H (2018). Development of a novel hybrid bioactive hydrogel for future clinical applications. J Biomater Appl.

[3] Fazel R, (ed.). Biomedical Engineering - Frontiers and Challenges [Internet]. InTech; 2011. Available from: http://www.intechopen.com/books/biomedical-engineering-frontiers-and-challenges.

[4] Jabbari E, Leijten J, Xu Q, Khademhosseini A (2016). The matrix reloaded: the evolution of regenerative hydrogels. Mater Today.

[5] H. Gulrez SK, Al-Assaf S, O G. Hydrogels: Methods of Preparation, Characterisation and Applications. In: Carpi A, (ed.). Progress in Molecular and Environmental Bioengineering - From Analysis and Modeling to Technology Applications [Internet]. InTech; 2011. Available from: http://www.intechopen.com/books/progress-in-molecular-and-environmental-bioengineering-from-analysis-and-modeling-to-technology-applications/hydrogels-methods-of-preparation-characterisation-and-applications.

[6] Van Vlierberghe S, Dubruel P, Schacht E (2011). Biopolymer-based hydrogels as scaffolds for tissue engineering applications: a review. Biomacromolecules..

[7] Ottenbrite Raphael M., Park Kinam, Okano Teruo (2010). Biomedical Applications of Hydrogels Handbook.

[8] Gerlach Gerald, Arndt Karl-Friedrich (2010). Hydrogel Sensors and Actuators.

[9] Sathaye S, Mbi A, Sonmez C, Chen Y, Blair DL, Schneider JP (2015). Rheology of peptide- and protein-based physical hydrogels: are everyday measurements just scratching the surface?. WIREs Nanomed Nanobiotechnol.

[10] Khetan S, Guvendiren M, Legant WR, Cohen DM, Chen CS, Burdick JA (2013). Degradation-mediated cellular traction directs stem cell fate in covalently crosslinked three-dimensional hydrogels. Nat Mater.

[11] Hudalla GA, Eng TS, Murphy WL (2008). An approach to modulate degradation and mesenchymal stem cell behavior in poly(ethylene glycol) networks. Biomacromolecules..

[12] Vieira S, da Silva Morais A, Silva-Correia J, Oliveira JM, Reis RL. Natural-based hydrogels: from processing to applications. In: Encyclopedia of polymer science and technology. Hoboken, NJ, USA: John Wiley & Sons, Inc.; 2017. p. 1–27. http://doi.wiley.com/10.1002/0471440264.pst652.

[13] Liu X, Zheng C, Luo X, Wang X, Jiang H (2019). Recent advances of collagen-based biomaterials: multi-hierarchical structure, modification and biomedical applications. Mater Sci Eng: C.

[14] Achilli M, Lagueux J, Mantovani D (2010). On the effects of UV-C and pH on the mechanical behavior, molecular conformation and cell viability of collagen-based scaffold for vascular tissue engineering. Macromol Biosci.

[15] Couet F, Rajan N, Mantovani D (2007). Macromolecular biomaterials for scaffold-based vascular tissue engineering. Macromol Biosci.

[16] Marelli B, Achilli M, Alessandrino A, Freddi G, Tanzi MC, Farè S (2012). Collagen-reinforced electrospun silk fibroin tubular construct as small calibre vascular graft. Macromol Biosci.

[17] Pankajakshan D, Agrawal DK (2010). Scaffolds in tissue engineering of blood vessels. Can J Physiol Pharmacol.

[18] Lian J, Mansel BW, Ingham B, Prabakar S, Williams MAK (2017). Controlling chain flexibility in collagen networks to produce hydrogels with distinct properties. Soft Mater.

[19] Stegemann JP, Nerem RM (2003). Altered response of vascular smooth muscle cells to exogenous biochemical stimulation in two- and three-dimensional culture. Exp Cell Res.

[20] Vrana NE, Elsheikh A, Builles N, Damour O, Hasirci V (2007). Effect of human corneal keratocytes and retinal pigment epithelial cells on the mechanical properties of micropatterned collagen films. Biomaterials..

[21] Antman-Passig M, Shefi O (2016). Remote magnetic orientation of 3D collagen hydrogels for directed neuronal regeneration. Nano Lett.

[22] Lin K, Zhang D, Macedo MH, Cui W, Sarmento B, Shen G (2019). Advanced collagen-based biomaterials for regenerative biomedicine. Adv Funct Mater.

[23] Nair P, Thottappillil N. Scaffolds in vascular regeneration: current status. Vasc Health Risk Manag. 2015;79–91. 10.2147/VHRM.S50536 PMC4304530 25632236

[24] Bobryshev YV (2005). Calcification of elastic fibers in human atherosclerotic plaque. Atherosclerosis..

[25] Khavandgar Z, Roman H, Li J, Lee S, Vali H, Brinckmann J (2014). Elastin haploinsufficiency impedes the progression of arterial calcification in MGP-deficient mice. J Bone Min Res.

[26] Pai A, Leaf EM, El-Abbadi M, Giachelli CM (2011). Elastin degradation and vascular smooth muscle cell phenotype change precede cell loss and arterial medial calcification in a uremic mouse model of chronic kidney disease. Am J Pathol.

[27] Daamen WF, Hafmans T, Veerkamp JH, van Kuppevelt TH (2001). Comparison of five procedures for the purification of insoluble elastin. Biomaterials..

[28] Testera AM, Girotti A, de Torre IG, Quintanilla L, Santos M, Alonso M (2015). Biocompatible elastin-like click gels: design, synthesis and characterization. J Mater Sci Mater Med.

[29] Mithieux SM, Rasko JEJ, Weiss AS (2004). Synthetic elastin hydrogels derived from massive elastic assemblies of self-organized human protein monomers. Biomaterials..

[30] Boland ED, Matthews JA, Pawlowski KJ, Simpson DG, Wnek GE, Bowlin GL (2004). Electrospinning collagen and elastin: preliminary vascular tissue engineering. Front Biosci.

[31] Lamme EN, van Leeuwen RTJ, Jonker A, van Marle J, Middelkoop E (1998). Living skin substitutes: survival and function of fibroblasts seeded in a dermal substitute in experimental wounds. J Investigative Dermatol.

[32] Annabi N, Mithieux SM, Boughton EA, Ruys AJ, Weiss AS, Dehghani F (2009). Synthesis of highly porous crosslinked elastin hydrogels and their interaction with fibroblasts in vitro. Biomaterials..

[33] Thomas V, Zhang X, Catledge SA, Vohra YK (2007). Functionally graded electrospun scaffolds with tunable mechanical properties for vascular tissue regeneration. Biomed Mater.

[34] Catto V, Farè S, Freddi G, Tanzi MC (2014). Vascular tissue engineering: recent advances in small diameter blood vessel regeneration. ISRN Vasc Med.

[35] Seifu DG, Purnama A, Mequanint K, Mantovani D (2013). Small-diameter vascular tissue engineering. Nature Reviews. Cardiology..

[36] Grassl ED, Oegema TR, Tranquillo RT (2002). Fibrin as an alternative biopolymer to type-I collagen for the fabrication of a media equivalent. J Biomed Mater Res.

[37] Kim S-J, Jang J-D, Lee S-K (2007). Treatment of long tubular bone defect of rabbit using autologous cultured osteoblasts mixed with fibrin. Cytotechnology..

[38] Chrobak MO, Hansen KJ, Gershlak JR, Vratsanos M, Kanellias M, Gaudette GR (2017). Design of a fibrin microthread-based composite layer for use in a cardiac patch. ACS Biomater Sci Eng.

[39] Arun Kumar R, Sivashanmugam A, Deepthi S, Bumgardner JD, Nair SV, Jayakumar R (2016). Nano-fibrin stabilized CaSO 4 crystals incorporated injectable chitin composite hydrogel for enhanced angiogenesis & osteogenesis. Carbohydr Polym.

[40] Briganti E, Spiller D, Mirtelli C, Kull S, Counoupas C, Losi P (2010). A composite fibrin-based scaffold for controlled delivery of bioactive pro-angiogenetic growth factors. J Controlled Release.

[41] Kozlov PV, Burdygina GI (1983). The structure and properties of solid gelatin and the principles of their modification. Polymer..

[42] Sadeghi M, Heidari B (2011). Crosslinked graft copolymer of methacrylic acid and gelatin as a novel hydrogel with pH-responsiveness properties. Materials..

[43] Sajkiewicz P, Kołbuk D (2014). Electrospinning of gelatin for tissue engineering—molecular conformation as one of the overlooked problems. J Biomater Sci Polym Ed.

[44] Kuo W-T, Huang H-Y, Chou M-J, Wu M-C, Huang Y-Y (2011). Surface modification of gelatin nanoparticles with polyethylenimine as gene vector. J Nanomater.

[45] Bakhsheshi-Rad HR, Hadisi Z, Hamzah E, Ismail AF, Aziz M, Kashefian M (2017). Drug delivery and cytocompatibility of ciprofloxacin loaded gelatin nanofibers-coated Mg alloy. Mater Lett.

[46] Nagarajan S, Belaid H, Pochat-Bohatier C, Teyssier C, Iatsunskyi I, Coy E (2017). Design of boron nitride/gelatin electrospun nanofibers for bone tissue engineering. ACS Appl Mater Interfaces.

[47] Nagarajan S, Soussan L, Bechelany M, Teyssier C, Cavaillès V, Pochat-Bohatier C (2016). Novel biocompatible electrospun gelatin fiber mats with antibiotic drug delivery properties. J Mater Chem B.

[48] Nagarajan S, Pochat-Bohatier C, Teyssier C, Balme S, Miele P, Kalkura N (2016). Design of graphene oxide/gelatin electrospun nanocomposite fibers for tissue engineering applications. RSC Adv.

[49] Thomas LV, Nair PD. Influence of mechanical stimulation in the development of a medial equivalent tissue-engineered vascular construct using a gelatin-g-vinyl acetate co-polymer scaffold. J Biomater Sci Polym Ed. 2012. 10.1163/092050611X607148 22104760

[50] Panzavolta S, Gioffrè M, Focarete ML, Gualandi C, Foroni L, Bigi A (2011). Electrospun gelatin nanofibers: optimization of genipin cross-linking to preserve fiber morphology after exposure to water. Acta Biomater.

[51] Vepari C, Kaplan DL (2007). Silk as a biomaterial. Prog Polym Sci.

[52] Kim HJ, Kim MK, Lee KH, Nho SK, Han MS, Um IC (2017). Effect of degumming methods on structural characteristics and properties of regenerated silk. Int J Biol Macromol.

[53] Han F, Liu S, Liu X, Pei Y, Bai S, Zhao H (2014). Woven silk fabric-reinforced silk nanofibrous scaffolds for regenerating load-bearing soft tissues. Acta Biomater.

[54] Kim SH, Yeon YK, Lee JM, Chao JR, Lee YJ, Seo YB (2018). Precisely printable and biocompatible silk fibroin bioink for digital light processing 3D printing. Nat Commun.

[55] Wang Qiusheng, Han Guocong, Yan Shuqin, Zhang Qiang (2019). 3D Printing of Silk Fibroin for Biomedical Applications. Materials.

[56] Huang Z-M, Zhang YZ, Ramakrishna S, Lim CT (2004). Electrospinning and mechanical characterization of gelatin nanofibers. Polymer..

[57] Bencherif SA, Srinivasan A, Horkay F, Hollinger JO, Matyjaszewski K, Washburn NR (2008). Influence of the degree of methacrylation on hyaluronic acid hydrogels properties. Biomaterials..

[58] Remuzzi A, Mantero S, Colombo M, Morigi M, Binda E, Camozzi D (2004). Vascular smooth muscle cells on hyaluronic acid: culture and mechanical characterization of an engineered vascular construct. Tissue Eng.

[59] Zavan B, Vindigni V, Lepidi S, Iacopetti I, Avruscio G, Abatangelo G (2008). Neoarteries grown in vivo using a tissue-engineered hyaluronan-based scaffold. FASEB J.

[60] Prestwich GD (2011). Hyaluronic acid-based clinical biomaterials derived for cell and molecule delivery in regenerative medicine. J Control Release.

[61] Burdick JA, Prestwich GD (2011). Hyaluronic acid hydrogels for biomedical applications. Adv Mater.

[62] Draget KI, Smidsrød O, Skjåk-Bræk G. Alginates from Algae. In: Steinbüchel A (ed.) Biopolymers Online. Weinheim, Germany: Wiley-VCH Verlag GmbH & Co. KGaA; 2005. http://doi.wiley.com/10.1002/3527600035.bpol6008.

[63] Draget KI, Taylor C (2011). Chemical, physical and biological properties of alginates and their biomedical implications. Food Hydrocoll.

[64] Brandl F, Sommer F, Goepferich A (2007). Rational design of hydrogels for tissue engineering: Impact of physical factors on cell behavior. Biomaterials..

[65] Huang Y, Onyeri S, Siewe M, Moshfeghian A, Madihally SV (2005). In vitro characterization of chitosan–gelatin scaffolds for tissue engineering. Biomaterials..

[66] Hamedi H, Moradi S, Hudson SM, Tonelli AE (2018). Chitosan based hydrogels and their applications for drug delivery in wound dressings: a review. Carbohydr Polym.

[67] Zhang L, Ao Q, Wang A, Lu G, Kong L, Gong Y (2006). A sandwich tubular scaffold derived from chitosan for blood vessel tissue engineering. J Biomed Mater Res A.

[68] Chupa JM, Foster AM, Sumner SR, Madihally SV, Matthew HW (2000). Vascular cell responses to polysaccharide materials: in vitro and in vivo evaluations. Biomaterials..

[69] Madihally SV, Matthew HW (1999). Porous chitosan scaffolds for tissue engineering. Biomaterials..

[70] Bernkop-Schnürch A, Dünnhaupt S (2012). Chitosan-based drug delivery systems. Eur J Pharm Biopharm.

[71] Kawecki M, Łabuś W, Klama-Baryla A, Kitala D, Kraut M, Glik J (2018). A review of decellurization methods caused by an urgent need for quality control of cell-free extracellular matrix’ scaffolds and their role in regenerative medicine. J Biomed Mater Res Part B Appl Biomater.

[72] Crapo PM, Gilbert TW, Badylak SF (2011). An overview of tissue and whole organ decellularization processes. Biomaterials..

[73] Wagenseil JE, Mecham RP (2009). Vascular extracellular matrix and arterial mechanics. Physiological Rev.

[74] Guruswamy Damodaran R, Vermette P. Tissue and organ decellularization in regenerative medicine. Biotechnol Prog. 2018. http://doi.wiley.com/10.1002/btpr.2699. 10.1002/btpr.2699 30294883

[75] Wight TN, Kinsella MG, Qwarnström EE (1992). The role of proteoglycans in cell adhesion, migration and proliferation. Curr Opin Cell Biol.

[76] Pang X, Lin L, Tang B. Unraveling the role of calcium ions in the mechanical properties of individual collagen fibrils. Sci Rep. 2017;7. http://www.nature.com/articles/srep46042. 10.1038/srep46042 PMC5380965 28378770

[77] Saldin LT, Cramer MC, Velankar SS, White LJ, Badylak SF (2017). Extracellular matrix hydrogels from decellularized tissues: structure and function. Acta Biomaterialia.

[78] Ganji F, Abdekhodaie MJ, Ramazani SAA (2007). Gelation time and degradation rate of chitosan-based injectable hydrogel. J Sol–Gel Sci Technol.

[79] Cho J, Heuzey M-C, Bégin A, Carreau PJ (2005). Physical gelation of chitosan in the presence of β-glycerophosphate: the effect of temperature. Biomacromolecules..

[80] Yamaoka H, Asato H, Ogasawara T, Nishizawa S, Takahashi T, Nakatsuka T (2006). Cartilage tissue engineering using human auricular chondrocytes embedded in different hydrogel materials. J Biomed Mater Res Part A.

[81] Kuijpers AJ, Engbers GHM, Krijgsveld J, Zaat SAJ, Dankert J, Feijen J (2000). Cross-linking and characterisation of gelatin matrices for biomedical applications. J Biomater Sci Polym Ed.

[82] Zawko S, Suri S, Truong Q, Schmidt C (2009). Photopatterned anisotropic swelling of dual-crosslinked hyaluronic acid hydrogels. Acta Biomaterialia.

[83] Abbah SA, Lu WW, Chan D, Cheung KMC, Liu WG, Zhao F (2008). Osteogenic behavior of alginate encapsulated bone marrow stromal cells: an in vitro study. J Mater Sci Mater Med.

